# Impact of Counseling Received by Adolescents Undergoing Voluntary Medical Male Circumcision on Knowledge and Sexual Intentions

**DOI:** 10.1093/cid/cix973

**Published:** 2018-04-03

**Authors:** Michelle R Kaufman, Eshan U Patel, Kim H Dam, Zoe R Packman, Lynn M Van Lith, Karin Hatzold, Arik V Marcell, Webster Mavhu, Catherine Kahabuka, Lusanda Mahlasela, Emmanuel Njeuhmeli, Kim Seifert Ahanda, Getrude Ncube, Gissenge Lija, Collen Bonnecwe, Aaron A R Tobian

**Affiliations:** 1 Johns Hopkins Bloomberg School of Public Health, Baltimore, Maryland; 2 Department of Pathology, Johns Hopkins University School of Medicine, Baltimore, Maryland; 3 Johns Hopkins Center for Communication Programs, Baltimore, Maryland; 4 Population Services International, Harare, Zimbabwe; 5 Department of Pediatrics, Johns Hopkins University School of Medicine, Baltimore, Maryland; 6 Centre for Sexual Health and HIV/AIDS Research, Harare, Zimbabwe; 7 CSK Research Solutions, Dar es Salaam, Tanzania; 8 Centre for Communication Impact, Pretoria, South Africa; 9 Office of HIV/AIDS, Global Health Bureau, United States Agency for International Development, Washington, District of Columbia; 10 Ministry of Health and Child Care, Harare, Zimbabwe; 11 Ministry of Health, Community Development, Gender, Elderly and Children, Dar es Salaam, Tanzania; 12 National Department of Health, Pretoria, South Africa

**Keywords:** adolescents, voluntary medical male circumcision, HIV prevention, HIV counseling, sub-Saharan Africa

## Abstract

**Background:**

Little is known regarding the impact of counseling delivered during voluntary medical male circumcision (VMMC) services on adolescents’ human immunodeficiency virus (HIV) knowledge, VMMC knowledge, or post-VMMC preventive sexual intentions. This study assessed the effect of counseling on knowledge and intentions.

**Methods:**

Surveys were conducted with 1293 adolescent clients in 3 countries (South Africa, n = 299; Tanzania, n = 498; Zimbabwe, n = 496). Adolescents were assessed on HIV and VMMC knowledge-based items before receiving VMMC preprocedure counseling and at a follow-up survey approximately 10 days postprocedure. Sexually active adolescents were asked about their sexual intentions in the follow-up survey. Prevalence ratios (PRs) and 95% confidence intervals (CIs) were calculated by modified Poisson regression models with generalized estimating equations and robust variance estimators.

**Results:**

Regarding post-VMMC HIV prevention knowledge, older adolescents were significantly more likely than younger adolescents to know that a male should use condoms (age 10–14 years, 41.1%; 15–19 years, 84.2%; aPR, 1.38 [95% CI, 1.19–1.60]), have fewer sex partners (age 10–14 years, 8.1%; age 15–19 years, 24.5%; aPR, 2.10 [95% CI, 1.30–3.39]), and be faithful to one partner (age 10–14 years, 5.7%; age 15–19 years, 23.2%; aPR, 2.79 [95% CI, 1.97–3.97]) to further protect himself from HIV. Older adolescents demonstrated greater improvement in knowledge in most categories, differences that were significant for questions regarding number of sex partners (aPR, 2.01 [95% CI, 1.18–3.44]) and faithfulness to one partner post-VMMC (aPR, 3.28 [95% CI, 2.22–4.86]). However, prevention knowledge levels overall and HIV risk reduction sexual intentions among sexually active adolescents were notably low, especially given that adolescents had been counseled only 7–10 days prior.

**Conclusions:**

Adolescent VMMC counseling needs to be improved to increase knowledge and postprocedure preventive sexual intentions.

Voluntary medical male circumcision (VMMC) is a key human immunodeficiency virus (HIV) prevention intervention implemented in high-priority settings in eastern and southern Africa to slow HIV incidence among sexually active males [1–5]. The World Health Organization (WHO) and the US President’s Emergency Plan for AIDS Relief (PEPFAR) recommend that all VMMC patients, regardless of age, receive counseling that explains the link between VMMC and reduced HIV acquisition, emphasizes the need to abstain from sexual intercourse and/or masturbation during the healing period, and encourages increased knowledge on how to further protect oneself from HIV post-VMMC [6–8].

Despite WHO and PEPFAR guidance, little is known about the counseling content that male adolescents are exposed to as part of VMMC service delivery. Past work presented in this same supplement based on a study in Tanzania, South Africa, and Zimbabwe [9] showed that the counseling content presented during the VMMC process for adolescents is lacking many crucial elements, including how to prevent complications during the wound healing period, how to identify VMMC complications should they occur, and how to properly use condoms. Younger adolescents (10–14 years of age) were found to receive even fewer key components of counseling topics compared to older adolescents (15–19 years of age).

Also, it is unknown whether the brief counseling opportunity occurring immediately before and/or after the VMMC procedure has a measurable impact on adolescents’ HIV and VMMC knowledge or post-VMMC sexual intentions, or whether these outcomes vary by adolescent age group. While other research has shown that single-session HIV counseling with adults has the potential to result in reduced sexually transmitted infection (STI) incidence and unprotected sex [10], the impact of single-session VMMC-related counseling is unknown.

The aim of this study was to assess the effect of counseling on sexual knowledge and behavioral intention outcomes and to determine whether this varies by adolescent age group. Specifically, we aimed to assess adolescents’ (1) change in knowledge of the importance of avoiding sexual practices during the VMMC healing period; (2) change in knowledge of how VMMC affects HIV risk and how to further reduce risk post-VMMC; and (3) sexual intentions post-VMMC among those who were already sexually active before VMMC.

## METHODS

### Ethics Statement

The Human Sciences Research Council in South Africa, Tanzania National Institute for Medical Research, Medical Research Council of Zimbabwe, and Johns Hopkins Bloomberg School of Public Health Institutional Review Board approved the study prior to data collection.

### Settings and Participants

In collaboration with local investigators, federal ministries of health, and a technical advisory group, study sites were selected using a purposive cluster sampling design, as previously described [11]. Study participants (n = 1526) were adolescent male VMMC clients 10–19 years of age drawn from 14 VMMC sites across the 3 countries (South Africa, n = 446, 4 sites; Tanzania n = 540, 4 sites; Zimbabwe n = 540, 6 sites) from June 2015 to September 2016. Of the 1526 participants who received VMMC, 233 (15.3%) did not complete a follow-up survey (Supplementary Table 1). Loss to follow-up was greater in South Africa due to a local government election in the Orange Farm township that occurred during the data collection period. Associated community protests and restrictions on movement within the township prevented the research team from entering the area due to blockaded roads.

### Procedures

In collaboration with research staff, VMMC providers or community mobilizers recruited prospective adolescent male VMMC clients as study participants. Adolescents provided consent if aged 18–19 years or assent and parent/guardian permission if a minor. Baseline surveys were conducted just before the client received preprocedure counseling. Follow-up surveys were conducted approximately 7–10 days postprocedure during the adolescents’ follow-up clinic appointment or in their home if they did not attend the appointment. Trained data collectors administered the survey in a private area in the participants’ language of choice (Sesotho, isiZulu, or isiSwati in South Africa; kiSwahili in Tanzania; Shona or Ndebele in Zimbabwe).

### Measures

#### Demographics

Participants’ demographic information was obtained during the preprocedure survey. Data on the types of counseling received (individual and/or group formats; pre- and/or postprocedure) were obtained at the follow-up survey.

#### Voluntary Medical Male Circumcision and Human Immunodeficiency Virus Knowledge

Knowledge-based items were assessed before receiving VMMC preprocedure counseling and again at the follow-up survey 7–10 days postprocedure. Items included knowledge about VMMC postprocedure care and knowledge regarding further HIV prevention behaviors. Categorical items were coded as 1 (“correct response”) and 0 (“other”). Unprompted responses to “What should a male do to protect himself from HIV after circumcision?” were coded by trained interviewers into predetermined categories (Supplementary Table 2).

#### Sexual Intentions

Participants aged ≥13 years who reported they were sexually active (13–14 years, n = 14; 15–19 years, n = 147) were asked in the follow-up survey whether their sexual intentions had changed since VMMC. These items were examined through 5 Likert-style questions, such as “Now that you have been circumcised, will your condom use increase, decrease, or remain the same?”

### Statistical Analyses

Prevalence ratio (PRs) and 95% confidence intervals (CIs) were calculated by modified Poisson regression models with generalized estimating equations and robust variance estimators to account for clustering of responses at the facility level. Hypothesized confounding factors and those shown to have an association with the outcome in univariable models (*P* < .05) were included in the final multivariable model. Because the primary analysis only included adolescents who participated in a follow-up survey, a sensitivity analysis was conducted using multiple imputation by chained equations to account for loss to follow-up (n = 233/1526). Selected predictors were based on unadjusted associations with outcome variables; highly collinear predictors were dropped. Twenty imputations were conducted. All analyses were performed using Stata SE software version 14.2 (StataCorp, College Station, Texas).

## RESULTS

### Study Population and Demographics

Data from participants who completed both the initial and follow-up surveys were included in the primary analysis of this study (South Africa, n = 299; Tanzania, n = 498; Zimbabwe, n = 496). Older adolescents had greater loss to follow-up compared to younger adolescents (odds ratio, 1.42 [95% CI, 1.07–1.89]; Supplementary Table 1). Table 1 shows characteristics of the participants in the primary analysis by age group. Approximately half of the adolescents were from urban areas (53.8%). A minimal number of younger adolescents (10–14 years of age, 5.7%) reported ever having had sexual experience, while 37.4% of older adolescents (15–19 years) reported some sexual experience. Approximately one-quarter (25.7%) reported receiving only individual counseling during the VMMC service, 35.3% received only group counseling, and 38.3% received a combination of both. It was common for younger adolescents (10–14 years of age, 56.1%) to be accompanied by parents/guardians to the preprocedure counseling session, but this was rarer for older adolescents (15–19 years of age, 12.5%).

**Table 1.** Characteristics of the Study Population

Characteristic	Age 10–14 y	Age 15–19 y	Overall
	(n = 836)	(n = 457)	(N = 1293)
Country			
South Africa	187 (22.4)	112 (24.5)	299 (23.1)
Tanzania	413 (49.4)	85 (18.6)	498 (38.5)
Zimbabwe	236 (28.2)	260 (56.9)	496 (38.4)
Facility setting			
Urban	429 (51.3)	267 (58.4)	696 (53.8)
Periurban	149 (17.8)	43 (9.4)	192 (14.8)
Rural	258 (30.9)	147 (32.2)	405 (31.3)
Preprocedure counseling			
Individual only	139 (16.6)	193 (42.2)	332 (25.7)
Group only	282 (33.7)	175 (38.3)	457 (35.3)
Both	408 (48.8)	87 (19.0)	495 (38.3)
Parent/guardian attendance at preprocedure counseling session			
No	361 (43.2)	399 (87.3)	760 (58.8)
Yes	469 (56.1)	57 (12.5)	526 (40.7)
Received postprocedure counseling session			
No	656 (78.5)	233 (51.0)	889 (68.8)
Yes	180 (21.5)	224 (49.0)	404 (31.2)
Ever had sexual experience			
No	787 (94.1)	286 (62.6)	1073 (83.0)
Yes	48 (5.7)	171 (37.4)	219 (16.9)
Education			
No school	6 (0.7)	10 (2.2)	16 (1.2)
Some primary school	720 (86.1)	129 (28.2)	129 (10.0)
Completed primary school	110 (13.2)	258 (56.7)	368 (28.5)

Data are presented as n (%). Percentages may not add up to 100% due to missing data.

Abbreviation: VMMC, voluntary medical male circumcision.

### Age-Related Disparities in Voluntary Medical Male Circumcision and Human Immunodeficiency Virus Knowledge

**Figure 1.**
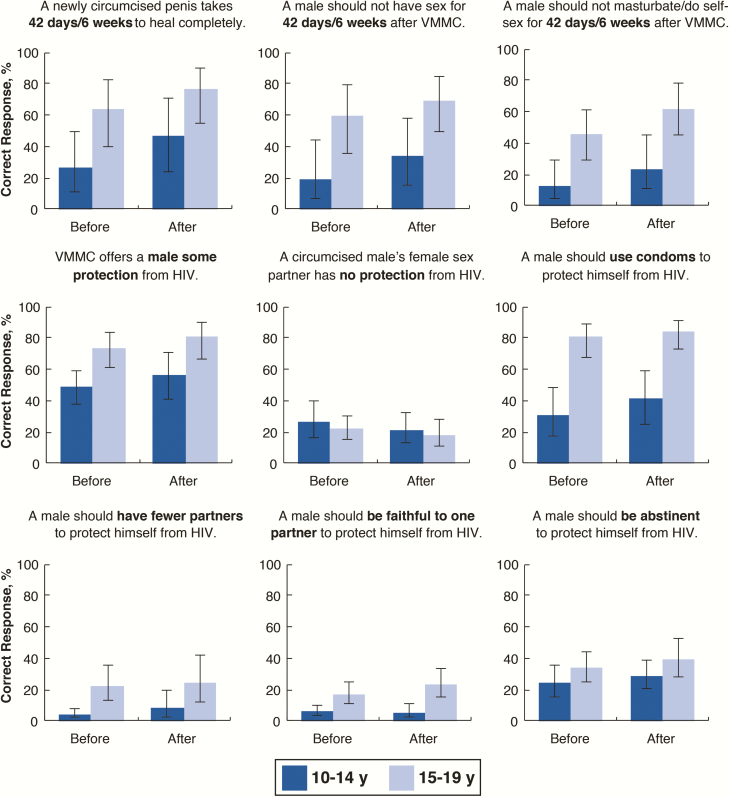
Voluntary medical male circumcision (VMMC) postprocedure care and human immunodeficiency virus (HIV) prevention knowledge before and after VMMC counseling, by age group. Bars represent the percent correctly answering a given question. Data are limited to complete cases who had available data in the pre- and post-procedure surveys. Error bars represent design-based 95% confidence intervals as estimated by Taylor series linearization and account for clustering at the facility level.

Knowledge levels about postprocedure care and post-VMMC HIV prevention in both the baseline and follow-up surveys were notably low for both age groups. They were consistently lower for younger adolescents compared to older adolescents and remained disparate even after VMMC counseling (Figure 1 and Table 2). In the follow-up survey, a significantly larger proportion of older adolescents answered seven of the nine items correctly compared with younger adolescents in the univariate analyses (Table 2).

**Table 2.** Age Differences in Voluntary Medical Male Circumcision (VMMC) and Human Immunodeficiency Virus Prevention Knowledge After VMMC Counseling

Correct Response	Age Group, y	PR (95% CI)	aPR (95% CI)^ a ^
VMMC postprocedure care knowledge			
A newly circumcised penis takes 42 d/6 wk to heal completely	10–14	Ref.	Ref.
	15–19	**1.24 (1.06–1.45**)	1.08 (.98–1.19)
A male should not have sex for 42 d/6 wk after VMMC	10–14	Ref.	Ref.
	15–19	**1.45 (1.12–1.87**)	1.14 (.98–1.32)
A male should not masturbate/do self-sex for 42 d/6 wk after VMMC	10–14	Ref.	Ref.
	15–19	**1.79 (1.30–2.48**)	1.38 (.99–1.91)
HIV prevention knowledge			
VMMC offers a male some protection from HIV	10–14	Ref.	Ref.
	15–19	**1.19 (1.06–1.33**)	1.10 (.98–1.24)
A circumcised male’s female sex partner has no protection from HIV	10–14	Ref.	Ref.
	15–19	0.99 (.75–1.32)	1.15 (.93–1.43)
A male should use condoms to protect himself from HIV^b^	10–14	Ref.	Ref.
	15–19	**1.71 (1.37–2.14**)	**1.38 (1.19–1.60**)
A male should have fewer partners to protect himself from HIV^b^	10–14	Ref.	Ref.
	15–19	**2.73 (1.46–5.11**)	**2.10 (1.30–3.39**)
A male should be faithful to one partner to protect himself from HIV^b^	10–14	Ref.	Ref.
	15–19	**3.07 (1.99–4.75**)	**2.79 (1.97–3.97**)
A male should be abstinent to protect himself from HIV^b^	10–14	Ref.	Ref.
	15–19	1.19 (.96–1.48)	1.24 (.93–1.67)

Prevalence ratios and 95% CIs of a correct response in the follow-up survey (see Figure 1) were calculated by modified Poisson regression models with generalized estimating equations and robust variance estimators to account for clustering of responses at the facility level. Estimates in bold have a *P* value <.05.

Abbreviations: aPR, adjusted prevalence ratio; CI, confidence interval; HIV, human immunodeficiency virus; PR, prevalence ratio; VMMC, voluntary medical male circumcision.

^a^The multivariable model for each response included adjustment for country, preprocedure counseling mode, receipt of postprocedure counseling session, parent/guardian attendance at counseling session, ever had a sexual experience, and education.

^b^Interviewers recorded unprompted free-response answers to “What should a male do to protect himself from HIV after circumcision?” Answers were recorded and coded into a predetermined list of categories. Relevant response categories are shown.

At follow-up, all 3 postprocedure care knowledge items showed significant age differences in the correct response in the unadjusted model, however, these differences were no longer significant when accounting for country, preprocedure counseling mode, receipt of postprocedure counseling session, parent/guardian attendance at the counseling session, ever having had a sexual experience, and education (*P* > .05; Table 2). For post-VMMC HIV prevention knowledge, older compared with younger adolescents were significantly more likely to know that following VMMC a male should use condoms (10–14 years of age, 41.1%; 15–19 years of age, 84.2%; aPR, 1.38 [95% CI, 1.19–1.60]), have fewer sex partners (10–14 years of age, 8.1%; 15–19 years of age, 24.5%; aPR, 2.10 [95% CI, 1.30–3.39]), and be faithful to one partner (10–14 years of age, 5.7%; 15–19 years of age, 23.2%; aPR, 2.79 [95% CI, 1.97–3.97]) to further protect himself from HIV, after controlling for other covariates (Table 2).

### Changes in Voluntary Medical Male Circumcision/Human Immunodeficiency Virus Knowledge Pre- to Postprocedure

Improvements in knowledge regarding VMMC postprocedure care and post-VMMC HIV prevention were greater in the univariate analyses among older adolescents who initially provided incorrect answers at baseline than younger adolescents who initially answered incorrectly at baseline (Table 3). While younger adolescents had less knowledge of a majority of the items in the follow-up survey (as noted in the previous section), an age difference in positive knowledge change (incorrect to correct) remained significant in the adjusted models for the items asking about the need for a male to have fewer sex partners (10–14 years of age, 6.4%; 15–19 years of age, 17.8%; aPR, 2.01 [95% CI, 1.18–3.44]) and to be faithful to one partner post-VMMC (10–14 years of age, 4.3%; 15–19 years of age, 17.8%; aPR, 3.28 [95% CI, 2.22–4.86]). (See Supplementary Tables 3 and 4 for sensitivity analyses of postprocedure knowledge and changes in knowledge using multiple imputation by chained equations to account for loss to follow-up. Both sensitivity analyses found results consistent with the primary analyses.)

**Table 3.** Age Differences in the Proportion of Adolescents Who Had an Improvement in Voluntary Medical Male Circumcision Knowledge and Human Immunodeficiency Virus Prevention Knowledge From Baseline to Follow-up

Correct Response	Age Group, y	% (no./No.)	PR (95% CI)	aPR (95% CI)^ a ^
VMMC postprocedure care knowledge				
A newly circumcised penis takes 42 d/6 wk to heal completely	10–14	31.8 (194/611)	Ref.	Ref.
	15–19	51.5 (85/165)	**1.42 (1.15–1.74**)	1.09 (.85–1.39)
A male should not have sex for 42 d/6 wk after VMMC	10–14	18.4 (111/604)	Ref.	Ref.
	15–19	41.8 (71/170)	**1.71 (1.38–2.13**)	1.10 (.80–1.52)
A male should not masturbate/do self- sex for 42 d/6 wk after VMMC	10–14	16.9 (123/726)	Ref.	Ref.
	15–19	42.0 (103/245)	**1.78 (1.31–2.41**)	1.26 (.82–1.92)
HIV prevention knowledge				
VMMC offers a male some protection from HIV	10–14	43.1 (185/429)	Ref.	Ref.
	15–19	48.3 (58/120)	0.97 (.78–1.19)	1.00 (.77–1.29)
A circumcised male’s female sex partner has no protection from HIV	10–14	12.4 (76/615)	Ref.	Ref.
	15–19	9.2 (33/357)	0.81 (.60–1.10)	1.16 (.82–1.65)
A male should use condoms to protect himself from HIV^b^	10–14	24.7 (143/580)	Ref.	Ref.
	15–19	51.7 (46/89)	**1.76 (1.35–2.28**)	1.10 (.86–1.41)
A male should have fewer partners to protect himself from HIV^b^	10–14	6.4 (51/799)	Ref.	Ref.
	15–19	17.8 (63/354)	**2.67 (1.33–5.36**)	**2.01 (1.18–3.44**)
A male should be faithful to one partner to protect himself from HIV^b^	10–14	4.3 (34/782)	Ref.	Ref.
	15–19	17.8 (67/377)	**3.24 (2.00–5.26**)	**3.28 (2.22–4.86**)
A male should be abstinent to protect himself from HIV^b^	10–14	19.6 (124/632)	Ref.	Ref.
	15–19	27.4 (83/303)	1.20 (.85–1.68)	1.31 (.84–2.06)

No. is the number of participants who had an incorrect response at baseline; the proportion shown indicates the participant cited the correct response in the follow-up survey, thereby showing an improvement in knowledge. Prevalence ratios and 95% CIs comparing the proportion who improved by age group were calculated by modified Poisson regression models with generalized estimating equations and robust variance estimators to account for clustering of responses at the facility level. Estimates in bold have a *P* value <.05.

Abbreviations: aPR, adjusted prevalence ratio; CI, confidence interval; HIV, human immunodeficiency virus; PR, prevalence ratio; VMMC, voluntary medical male circumcision.

^a^The multivariable model for each response included adjustment for country, preprocedure counseling mode, receipt of postprocedure counseling session, parent/guardian attendance at counseling session, ever had a sexual experience, and education.

^b^Interviewers recorded unprompted free-response answers to “What should a male do to protect himself from HIV after VMMC?” Answers were recorded and coded into predetermined list of categories. Relevant response categories are shown.

Table 4 presents factors related to improvement in the postprocedure follow-up survey for each VMMC knowledge and HIV prevention knowledge item from the baseline survey, including counseling format, parent/guardian attendance at the counseling, receipt of postprocedure counseling session, previous sexual experience, and education. For instance, parent/guardian attendance at the counseling session was significantly associated with a lower chance of improvement for five of the nine items (Table 4).

**Table 4.** Factors Related to Improved Voluntary Medical Male Circumcision Postprocedure Knowledge and Human Immunodeficiency Virus Prevention Knowledge From Baseline to Follow-up

Knowledge Statement	Group-Only Counseling (vs Individual-Only Counseling)	Individual and Group Counseling (vs Individual-Only Counseling)	Parent/Guardian Attendance at Counseling Session	Receipt of Postprocedure Counseling Session	Ever Had a Sexual Experience	Some Primary School (vs No Primary School)	Completed Primary School (vs No Primary School)
A newly circumcised penis takes 42 d/6 wk to heal completely	0.95 (.79–1.14)	**0.69 (.53–.90**)	**0.68 (.58–.81**)	**1.34 (1.03–1.76**)	**1.42 (1.18–1.72**)	1.09 (.70–1.69)	1.59 (.90–2.78)
A male should not have sex for 42 d/6 wk after VMMC	**0.76 (.59–.98**)	**0.59 (.43–.81**)	**0.50 (.31–.80**)	**0.51 (.40–.64**)	**1.44 (1.19–1.74**)	1.06 (.55–2.04)	1.97 (.93–4.17)
A male should not masturbate/do self-sex for 42 d/6 wk after VMMC	**0.77 (.61–.97**)	**0.48 (.34–.68**)	**0.45 (.36–.56**)	1.13 (.86–1.50)	**1.73 (1.39–2.15**)	1.12 (.51–2.45)	2.03 (.87–4.73)
VMMC offers a male some protection from HIV	1.47 (.88–2.45)	1.29 (.76–2.18)	1.11 (.85–1.44)	1.32 (.97–1.79)	**0.70 (.54–.92**)	1.45 (.36–5.80)	1.60 (.38–6.75)
A circumcised male’s female sex partner has no protection from HIV	0.72 (.36–1.43)	1.48 (.71–3.07)	1.45 (1.00–2.11)	**0.62 (.42–.92**)	0.76 (.49–1.17)	0.96 (.22–4.24)	0.62 (.14–2.83)
A male should use condoms to protect himself from HIV	**0.57 (.44–.73**)	**0.53 (.35–.81**)	**0.63 (.52–.76**)	1.18 (.78–1.77)	**2.05 (1.58–2.65**)	1.25 (.58–2.71)	2.20 (.96–5.04)
A male should have fewer partners to protect himself from HIV	0.70 (.19–2.63)	0.42 (.13–1.32)	**0.36 (.17–.76**)	**2.09 (1.43–3.05**)	1.68 (.89–3.17)	0.71 (.25–2.03)	1.63 (.44–6.04)
A male should be faithful to one partner to protect himself from HIV	**0.66 (.44–.97**)	0.60 (.30–1.22)	0.60 (.30–1.22)	1.70 (.75–3.87)	1.50 (.90–2.47)	**0.40 (.16–.98**)	0.71 (.23–2.20)
A male should be abstinent to protect himself from HIV	1.00 (.68–1.47)	0.98 (.56–1.71)	1.03 (.69–1.53)	**1.50 (1.09–2.06**)	0.74 (.45–1.20)	1.38 (.62–4.82)	**1.65 (.41–6.54**)

Data are shown as prevalence ratios and 95% confidence intervals, representing unadjusted associations calculated by modified Poisson regression models with generalized estimating equations and robust variance estimators to account for clustering of responses at the facility level. Estimates in bold have a *P* value <.05.

Abbreviations: HIV, human immunodeficiency virus; VMMC, voluntary medical male circumcision.

### Age Differences in Postprocedure Sexual Intentions

**Figure 2.**
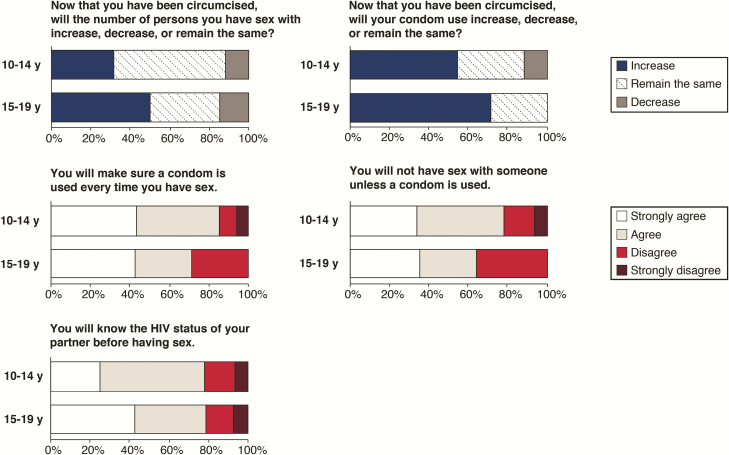
Sexual intentions after receiving voluntary medical male circumcision/human immunodeficiency virus (HIV) prevention counseling among 13- to 19-year-old males who had a previous sexual encounter of any kind (13–14 years, n = 14; 15–19 years, n = 147).

For all sexual intention items assessed in the follow-up survey, there were no major differences in responses from sexually active adolescents aged 13–14 years compared to those aged 15–19 years (Figure 2). A majority of older adolescents reported they intended to remain the same (56.5%) or decrease (11.6%) the number of their sex partners since receiving VMMC, whereas about half of younger adolescents (aged 13–14 years; 35.7% remain the same, 14.3% decrease) reported this. Both older (54.4%) and younger (71.4%) adolescents said they intended to increase their condom use post-VMMC. Many adolescents noted they “strongly agree” they will use a condom every time they have sex (13–14 years of age, 42.9%; 15–19 years of age, 43.5%), and that they will not have sex with someone unless a condom is used (13–14 years of age, 35.7%; 15–19 years of age, 34.0%) or without knowing a partner’s HIV status (13–14 years of age, 42.9%; 15–19 years of age, 25.3%).

## DISCUSSION

Older adolescents in this study had higher levels of knowledge of both post-VMMC procedure care and HIV prevention than did younger adolescents. Even for those items where there was no significant age difference in the adjusted models, knowledge overall was notably low, especially given the fact that the adolescents received counseling within the previous 10 days. Furthermore, HIV risk reduction sexual intentions at follow-up among those who were already sexually active at baseline were suboptimal. Not all sexually active adolescents intended to increase their condom use post-VMMC, less than half noted they would use a condom during every sex act, and only one-third said they would refuse sex with someone unless a condom is used. These results suggest that VMMC counseling for both sexually active and abstinent adolescents needs to be substantially improved to increase knowledge and post-VMMC preventive intentions against sexual risk.

To our knowledge, there are no other studies of the impact of adolescent VMMC-related counseling. In fact, a recent systematic review of studies addressing adolescent health service delivery for males in sub-Saharan Africa revealed a general absence of evidence-based services addressing the sexual and reproductive health needs of male adolescents, particularly for VMMC [12]. However, a few studies have examined whether HIV testing services results in reduced HIV risk outcomes for youth [13, 14]. One study in South Africa evaluated the impact of HIV testing services for youth (aged 15–24 years) on HIV incidence and found it had a protective effect, perhaps because youth are in less stable relationships and are more likely to engage with multiple sex partners, or because they have not yet formed sexual habits and are more amenable to counseling messages [13]. A meta-analysis of interventions to reduce sexual risk for HIV among adolescents in both developing and developed contexts found that multiple counseling sessions have the potential to reduce risky sexual behaviors and prevent STI transmission [15].

Single-session sexual risk reduction interventions in resource-constrained settings, which have been shown to encourage HIV preventive behaviors (such as increasing condom use), may be more feasible [16–18]. A study in South Africa found that brief, single-session HIV counseling with female and male adults had the potential to reduce the incidence of STIs and unprotected vaginal and anal intercourse, which could reduce subsequent HIV infections [10]. It is unknown whether this approach would be just as effective for adolescents. In the context of VMMC, risk reduction counseling for adolescents clearly requires further improvement to see the positive effects suggested in these other studies [19].

The current study and previous research begs the question, what is the true impact of the counseling delivered as part of the VMMC service, particularly for young males? Should program implementers use the VMMC service as a way to bring adolescents back for counseling focused solely on HIV risk reduction at the 1-week follow-up appointment or after the complete healing period has passed? If loss to follow-up is an issue, perhaps coupling VMMC more explicitly with community- or school-based HIV risk reduction interventions is needed. Packages of interventions that train health workers, focus on improving adolescent-friendly facilities and services, and provide out-of-facility intervention components may be more effective in low- and middle-income settings [20]. Such multilevel, multicomponent interventions, while difficult to implement, may be most effective at preventing HIV for future generations [19]. While VMMC is a unique opportunity to engage young males in sexual and reproductive health services [12, 21], this study found that the counseling provided to adolescents during the course of VMMC is insufficient. These findings are likely generalizable to other settings serving male adolescents in sub-Saharan Africa, as the study sites in the 3 countries reflect a range of geographic locations and ethnic backgrounds.

This study has limitations. Counseling received during a potentially anxiety-provoking experience may be less effective, particularly for younger males. Sexual intentions were only measured at follow-up, so it is unknown whether healthy intentions changed as a result of the counseling, remained static, or even decreased, after seeking VMMC. In addition, there may be selection bias, as only adolescents who completed the follow-up survey were included in this analysis. However, a sensitivity analysis using multiple imputation to account for adolescents lost to follow-up found nearly identical results.

There remains much room for improvement in the counseling for adolescents seeking VMMC services, particularly counseling designed to impact their knowledge and post-VMMC HIV risk reduction behaviors. The brief counseling opportunity provided during the service, while a starting point for guiding future healthy sexual behaviors, needs to be modified to enhance uptake of HIV risk reduction strategies.

## Supplementary Data

Supplementary materials are available at *Clinical Infectious Diseases* online. Consisting of data provided by the authors to benefit the reader, the posted materials are not copyedited and are the sole responsibility of the authors, so questions or comments should be addressed to the corresponding author.

Supplement1225_ZP

## Notes

***Acknowledgments.*** We are grateful to the adolescent participants in this study for sharing their perspectives and experiences. The authors also thank the VMMC facility managers, community mobilizers, and providers for their support. We acknowledge the contributions of the Technical Advisory Group for the adolescent VMMC assessment and their guidance throughout the study. We are privileged to work with this group of dedicated professionals from the US President’s Emergency Plan for AIDS Relief (PEPFAR), Centers for Disease Control and Prevention, United States Department of Defense, World Health Organization, United Nations Children’s Fund, and Bill & Melinda Gates Foundation. Thanks go to the Human Sciences Research Council in South Africa, CSK Research Solutions in Tanzania, PSI/Zimbabwe, and the Centre for Sexual Health and HIV/AIDS Research in Zimbabwe for their assistance with data collection. The authors also appreciate the assistance of Meaghen Murphy with copyediting and Maria Elena Figueroa with project startup.

***Financial support.*** This work was supported by USAID with PEPFAR funding (cooperative agreement number AID-OAA-A-12-00058) to the Johns Hopkins Center for Communication Programs and co-funded by the UK Department of International Development through the Integrated Support Program in Zimbabwe.

***Supplement sponsorship.*** This article appears as part of the supplement “Adolescent Voluntary Medical Male Circumcision: Vital Intervention Yet Improvements Needed,” sponsored by Johns Hopkins University.

***Potential conflicts of interest.*** All authors: No reported conflicts of interest. All authors have submitted the ICMJE Form for Disclosure of Potential Conflicts of Interest. Conflicts that the editors consider relevant to the content of the manuscript have been disclosed.
