# Supplementary material for: Impact of Counseling Received by Adolescents Undergoing Voluntary Medical Male Circumcision on Knowledge and Sexual Intentions
Source: Clin Infect Dis. 2018 Apr 3;66(Suppl 3):S221–8. doi: 10.1093/cid/cix973 (PMC5888933; doi:10.1093/cid/cix973)
Supplement: Supplement1225_ZP [file cix973_suppl_supplement1225_zp.docx]

**SUPPLEMENTARY INFORMATION.**

**Supplementary Table S1.** Characteristics of participants lost to follow-up.

|  | **Retained in Study** | | **Lost to Follow-Up** | | **OR (95% CI)** |
| --- | --- | --- | --- | --- | --- |
|  | **(n=1293)** | | **(n=233)** | |  |
| Age group |  |  |  |  |  |
| 10-14 years | 836 (64.7) | | 131 (56.2) | | Ref. |
| 15-19 years | 457 (35.3) | | 102 (43.8) | | **1.42 (1.07-1.89)** |
| Country |  |  |  |  |  |
| South Africa | 299 (23.1) | | 147 (63.1) | | Ref. |
| Tanzania | 498 (38.5) | | 42 (18.0) | | **0.17 (0.12-0.25)** |
| Zimbabwe | 496 (38.4) | | 44 (18.9) | | **0.18 (0.13-0.26)** |
| Facility Setting |  |  |  |  |  |
| Urban | 696 (53.8) | | 110 (47.2) | | Ref. |
| Peri-Urban | 192 (14.8) | | 59 (25.3) | | **1.94 (1.36-2.77)** |
| Rural | 405 (31.3) | | 64 (27.5) | | 1.00 (0.72-1.39) |
| Ever Had Sexual Experience |  |  |  |  |  |
| No | 1073 (83.0) | | 186 (79.8) | | Ref. |
| Yes | 219 (16.9) | | 44 (18.9) | | 1.16 (0.81-1.66) |
| Education |  |  |  |  |  |
| No school | 16 (1.2) | | 5 (2.1) | | Ref. |
| Some primary school | 907 (70.1) | | 121 (51.9) | | 0.43 (0.15-1.19) |
| Completed primary school | 368 (28.5) | | 107 (45.9) | | 0.93 (0.33-2.60) |
| Data are n (%).  Odds ratios (OR) were calculated from univariate logistic regression models. Estimates in bold have a *P* value <0.05. | | | | | |

**Supplementary Table S2.** Unprompted responses to “What should a male do to fully protect himself from HIV after VMMC?” by age group and category.

| **What should a male do to fully protect himself from HIV after VMMC?** | | | | | | |
| --- | --- | --- | --- | --- | --- | --- |
|  | Before VMMC Counseling | | | After VMMC Counseling | | |
|  | 10-14 | 15-19 | Total | 10-14 | 15-19 | Total |
|  | (967) | (559) | (1526) | (803) | (457) | (1260) |
| Use condoms | 322 (33.3) | 456 (81.6) | 778 (51.0) | 330 (41.1) | 385 (84.2) | 715 (56.7) |
| Have fewer partners | 43 (4.4) | 121 (21.6) | 164 (10.7) | 65 (8.1) | 112 (24.5) | 177 (14.0) |
| Be abstinent | 222 (23.0) | 185 (33.1) | 407 (26.7) | 233 (29.0) | 180 (39.4) | 413 (32.8) |
| Be faithful to one partner | 67 (6.9) | 99 (17.7) | 166 (10.9) | 46 (5.7) | 106 (23.2) | 152 (12.1) |
| Avoid/do not share sharp objects | 23 (2.4) | 9 (1.6) | 32 (2.1) | 27 (3.2) | 9 (2.0) | 36 (2.8) |
| Get tested for HIV | 16 (1.7) | 14 (2.5) | 30 (2.0) | 14 (1.7) | 15 (3.3) | 29 (2.2) |
| Sexual risk reduction strategies | 11 (1.1) | 3 (0.5) | 14 (0.9) | 17 (1.8) | 9 (1.6) | 26 (1.7) |
| Other | 24 (2.5) | 14 (2.5) | 38 (2.5) | 20 (2.4) | 10 (2.2) | 30 (2.3) |
| Don’t know | 359 (37.1) | 27 (4.8) | 386 (25.3) | 269 (32.2) | 18 (3.90) | 287 (22.2) |
| Data are n (%). | | | | | | |

**Supplementary Table S3.** Sensitivity analysis of VMMC post-procedure and HIV prevention knowledge *after* VMMC counseling by age group using multiple imputation to account for loss to follow-up.

| **Correct Response** | **Age Group, years** | **%** | **PR (95% CI)** | **aPR (95% CI)** |
| --- | --- | --- | --- | --- |
| A newly circumcised penis takes **42 days/6weeks** to heal completely. | 10-14 | 48.0% | Ref. | Ref. |
|  | 15-19 | 76.7% | **1.24 (1.07-1.44)** | 1.07 (0.97-1.18) |
| A male should not have sex for **42 days/6 weeks** after VMMC. | 10-14 | 34.6% | Ref. | Ref. |
|  | 15-19 | 69.7% | **1.45 (1.15-1.84)** | 1.13 (0.98-1.30) |
| A male should not masturbate/do self-sex **for 42 days/6 weeks** after VMMC. | 10-14 | 24.7% | Ref. | Ref. |
|  | 15-19 | 61.7% | **1.78 (1.32-2.40)** | **1.34 (1.01-1.78)** |
| VMMC offers a male **some protection** from HIV. | 10-14 | 55.6% | Ref. | Ref. |
|  | 15-19 | 79.8% | **1.20 (1.07-1.34)** | 1.11 (0.99-1.24) |
| A circumcised male's female sex partner has **no protection** from HIV. | 10-14 | 21.3% | Ref. | Ref. |
|  | 15-19 | 18.4% | 0.98 (0.76-1.27) | 1.11 (0.86-1.45) |
| A male should **use condoms** to protect himself from HIV. | 10-14 | 41.2% | Ref. | Ref. |
|  | 15-19 | 84.3% | **1.69 (1.37-2.08)** | **1.38 (1.20-1.58)** |
| A male should have **fewer partners** to protect himself from HIV. | 10-14 | 7.7% | Ref. | Ref. |
|  | 15-19 | 23.5% | **2.53 (1.43-4.48)** | **1.95 (1.27-3.02)** |
| A male should **be** **faithful to one partner** to protect himself from HIV. | 10-14 | 5.4% | Ref. | Ref. |
|  | 15-19 | 22.2% | **3.10 (2.05-4.70)** | **2.91 (1.97-4.29)** |
| A male should **be abstinent** to protect himself from HIV. | 10-14 | 28.0% | Ref. | Ref. |
|  | 15-19 | 39.2% | 1.19 (0.96-1.48) | 1.25 (0.96-1.64) |
| Prevalence ratios (PR) and 95% confidence intervals (CI) were calculated by modified Poisson regression models with generalized estimating equations and robust variance estimators to account for clustering of responses at the facility level. Estimates in bold have a *P* value <0.05.  Imputation model includes age, country, socioeconomic status, facility area, facility governance, parent attendance at counseling session, receipt of post-procedure counseling, having ever had a sexual experience, education, and pre-procedure counseling mode. Multiple imputation by chained equations (m=20) used.  ^1^ Multivariable model included adjustment for country, pre-procedure counseling mode, receipt of post-procedure counseling, parent attendance at counseling session, ever had a sexual experience, and education.  ^a^ Interviewers recorded unprompted free-response answers to "What should a male do to protect himself from HIV after circumcision?" Answers were recorded and coded into pre-determined list of categories. Relevant response categories are shown. | | | | |

**Supplementary Table S4.** Sensitivity analysis of positive changes to VMMC post-procedure knowledge and HIV prevention knowledge questions following VMMC counseling, using multiple imputation to account for loss to follow-up.

| **Correct Response** | **Age Group, years** | **%** | **PR (95% CI)** | **aPR (95% CI)** |
| --- | --- | --- | --- | --- |
| A newly circumcised penis takes **42 days/6weeks** to heal completely. | 10-14 | 32.4% | Ref. | Ref. |
|  | 15-19 | 51.9% | **1.44 (1.15-1.80)** | 1.13 (0.86-1.47) |
| A male should not have sex for **42 days/6 weeks** after VMMC. | 10-14 | 20.2% | Ref. | Ref. |
|  | 15-19 | 42.9% | **1.66 (1.30-2.11)** | 1.12 (0.83-1.53) |
| A male should not masturbate/do self-sex **for 42 days/6 weeks** after VMMC. | 10-14 | 17.9% | Ref. | Ref. |
|  | 15-19 | 42.1% | **1.76 (1.29-2.39)** | 1.25 (0.84-1.85) |
| VMMC offers a male **some protection** from HIV. | 10-14 | 43.9% | Ref. | Ref. |
|  | 15-19 | 47.5% | 0.95 (0.78-1.15) | 0.97 (0.75-1.26) |
| A circumcised male's female sex partner has **no protection** from HIV. | 10-14 | 12.5% | Ref. | Ref. |
|  | 15-19 | 9.5% | 0.80 (0.57-1.11) | 1.08 (0.71-1.64) |
| A male should **use condoms** to protect himself from HIV. | 10-14 | 25.8% | Ref. | Ref. |
|  | 15-19 | 53.0% | **1.74 (1.33-2.26)** | 1.17 (0.90-1.51) |
| A male should have **fewer partners** to protect himself from HIV. | 10-14 | 6.6% | Ref. | Ref. |
|  | 15-19 | 17.6% | **2.49 (1.33-4.65)** | **1.96 (1.17-3.28)** |
| A male should **be** **faithful to one partner** to protect himself from HIV. | 10-14 | 4.6% | Ref. | Ref. |
|  | 15-19 | 17.3% | **3.09 (1.96-4.86)** | **3.29 (2.20-4.92)** |
| A male should **be abstinent** to protect himself from HIV. | 10-14 | 20.3% | Ref. | Ref. |
|  | 15-19 | 28.0% | 1.20 (0.86-1.68) | 1.34 (0.87-2.06) |
| Prevalence ratios (PR) and 95% confidence intervals (CI) were calculated by modified Poisson regression models with generalized estimating equations and robust variance estimators to account for clustering of responses at the facility level. Estimates in bold have a *P* value <0.05. N is number of participants who had an incorrect response at baseline.  Imputation model includes age, country, socioeconomic status, facility area, facility governance, parent attendance at counseling session, receipt of post-procedure counseling, having ever had a sexual experience, education, and pre-procedure counseling mode. Multiple imputation by chained equations (m=20) used.  ^1^ Multivariable model included adjustment for country, pre-procedure counseling mode, receipt of post-procedure counseling, parent attendance at counseling session, ever had a sexual experience, and education.  ^a^ Interviewers recorded unprompted free-response answers to "What should a male do to protect himself from HIV after VMMC?" Answers were recorded and coded into pre-determined list of categories. Relevant response categories are shown. | | | | |
